# Why are poorer children at higher risk of obesity and overweight? A UK cohort study

**DOI:** 10.1093/eurpub/ckv219

**Published:** 2015-12-11

**Authors:** Alice Goisis, Amanda Sacker, Yvonne Kelly

**Affiliations:** 1 Department of Epidemiology & Public Health, University College London, 1-19 Torrington Place, London, WC1E 6BT, UK; 2 Department of Social Policy, London School of Economics, Houghton Street, London, WC2A 2AE, UK

## Abstract

**Background:** There is limited evidence on which risk factors attenuate income inequalities in child overweight and obesity; whether and why these inequalities widen as children age. **Method:** Eleven thousand nine hundred and sixty five singletons had complete data at age 5 and 9384 at age 11 from the Millennium Cohort Study (UK). Overweight (age 5 : 15%; age 11 : 20%) and obesity (age 5 : 5%; age 11 : 6%) were defined using the International Obesity Taskforce body mass index cut-points. To measure socioeconomic inequalities, we used quintiles of family income and as risk factors, we considered markers of maternal health behaviours and of children’s physical activity, sedentary behaviours and diet. Binary and multinomial logistic regression models were used. **Results:** The unadjusted analyses revealed stark income inequalities in the risk of obesity at age 5 and 11. At age 5, children in the bottom income quintile had 2.0 (95% CI: 1.4–2.8) increased relative risk of being obese whilst at age 11 they had 3.0 (95% CI: 2.0–4.5) increased risk compared to children in the top income quintile. Similar income inequalities in the risk of overweight emerged by age 11. Physical activity and diet were particularly important in explaining inequalities. Income inequalities in obesity and overweight widened significantly between age 5 and 11 and a similar set of risk factors protected against upward and promoted downward movements across weight categories. **Conclusions:** To reduce income inequalities in overweight and obesity and their widening across childhood the results support the need of early interventions which take account of multiple risk factors.

## Introduction

In recent decades, the prevalence of obesity among children and adults has increased dramatically in the UK and elsewhere.^1–3^ Obesity is linked to the development of numerous chronic diseases and poses significant health and economic burdens.^4^^,^^5^ In the context of lifelong health, we know that children who become overweight and obese are at higher risk of obesity throughout their lives.^6^^,^^7^ There is also evidence that overweight and obese children face higher risks of negative economic and social outcomes both in childhood and adulthood.^4^^,^^8^ Therefore, intervening early to reduce the prevalence of obesity and overweight could have long-term knock on effects for population health.

Numerous studies show that in many developed countries, including the UK, there are income and other socioeconomic inequalities in the risk of childhood obesity and overweight, and that these begin in the preschool years.^9–13^ A few studies also reveal that socioeconomic inequalities in overweight/obesity widen across childhood.^14^^,^^15^ These inequalities are likely explained by differential access to resources and/or knowledge by poorer parents who may practice worse health behaviours,^16–18^ but until now there has been no attempt to examine and compare several potential mechanisms linking family socioeconomic position to overweight/obesity in children. Our understanding of why children from socially disadvantaged backgrounds experience higher risk of obesity and overweight—and increasingly so across childhood—is limited.^10^^,^^11^^,^^19^

Our study, which focuses on the UK context, addresses this gap in knowledge in several ways. First, we examine which markers of family health behaviours and environmental risk factors reduce income inequalities in child overweight and obesity in the UK. To investigate whether influences are similar across childhood, we considered two age points: in early childhood at 5 years and on the cusp of adolescence at 11 years. Second, we investigate whether income inequalities widen across childhood by assessing whether poorer children were more likely to experience upward movements and less likely to experience downward movements across weight categories (normal weight, overweight, obese) between age 5 and 11. Third, we investigate which risk factors protect against upward movements and which promote downward movements across weight categories. To conduct the analyses, we used data from the large population-based UK Millennium Cohort Study.

## Methods

### The Millennium Cohort Study

The Millennium Cohort Study (MCS) is a UK nationally representative prospective cohort study of children born into 19 244 families.^20^ The sample population was drawn from all live births in the UK from September 2000 to January 2002. The sample was selected from a random sample of electoral wards with a stratified sampling strategy to ensure adequate representation of all four UK countries, and of disadvantaged and ethnically diverse areas. The first sweep of data was collected when cohort members were around 9 months and the subsequent four sweeps of data were collected at ages 3, 5, 7 and 11 years. During home visits interviews were conducted and questions asked about socioeconomic circumstances, health-related behaviours including smoking during pregnancy, infant feeding, physical and sedentary activities and dietary markers. Interview data were available for 79 and 69% of families when cohort members were aged 5 and 11, respectively.

### Child overweight and obesity

Overweight and obesity were defined using the International Obesity Taskforce (IOTF) body mass index (kg m^−^^2^) cut-points^21^ which are age and gender specific. At ages 5 and 11, children were weighed without shoes or outdoor clothing using Tanita HD-305 scales (Tanita UK Ltd., Middlesex, UK) and weights were recorded in kilograms to one decimal place. Heights were obtained using the Leicester Height Measure Stadiometer (Seca Ltd., Birmingham, UK) and recorded to the nearest millimetre.^22^

### Family income

To measure socioeconomic inequalities, we used quintiles of family income after adjustment using the modified OECD scale, which were derived by the MCS survey team.^20^ This scale takes into account the number of people in the household by setting the family’s needs relative to those of a couple with no children.^20^^,^^23^ Family income was used in the analyses as a marker of access to resources (to purchase ‘healthy’ food, organized physical activities and to live in safer neighbourhoods etc.), and we expected children from poorer families to be at increased risk of overweight or obesity.^24^ In the analyses, the top income quintile was the reference category.

### Potentially modifiable risk factors

We selected markers of health behaviours and environmental influences hypothesized to attenuate income inequalities in child overweight and obesity. To capture the mother’s health-related behaviours during pregnancy and after birth, we considered whether the mother smoked during pregnancy,^25^ duration of breastfeeding (no breastfeeding, up to 2 months, 2–4 months, more than 4 months)^26^^,^^27^ and whether the child was introduced to solid foods before 4 months of age.^24^^,^^28^ Markers of children’s physical activity and sedentary behaviours were frequency of sport/exercise (less than once, once or twice, three times or more per week),^29^ active playing with a parent (less than once, once or twice, three times or more per week), hours watching TV (less than one, between one and three, three or more per day),^18^ hours playing on a PC (less than one, between one and three, three or more per day),^30^ at least weekly use of a playground (at age 5), whether there was a playground in the area (age 11), journeys in the area by bike (measure available at age 11 only) and weekday bedtime (at age 5: before 7:30, 7:30–7:59, 8–8:59, 9 or later, no regular bedtime; at age 11: before 8, 8–8:59, 9–9:59, 10 or later, no regular bedtime).^18^ A marker of active transport—whether children actively commuted to school—was excluded as this was not significantly associated with adiposity. Markers of the dietary environment in which children were growing up were whether the child skipped breakfast,^31^ fruit consumption (none or one, two, three or more per day),^9^ sweet drinks consumption (at age 5: as main drink in between meals; at age 11: at least once a day)^32^ and maternal BMI (obtained using mothers’ self-reported weight and height).^33^ Data on early life markers of maternal health behaviours were collected when cohort members were aged around 9 months (Sweep 1) and the other risk factors were, unless indicated otherwise, measured contemporaneously i.e. at ages 5 or 11 years. Children’s health behaviours, at both ages, were reported by the mother at interview.

### Inclusion criteria, exclusionsand losses to follow up

We dropped observations with missing values on adiposity and risk factors (at age 5 = 3104; at age 11 = 3703). From the complete case sample, we dropped children who had implausible (*z*-score was ≤−5 or ≥5) BMI values (at age 5 *n* = 14; at age 11 *n* = 2). If the mother was pregnant when the cohort member was aged 5 or 11 we used mother’s BMI recorded at the previous Sweep of data collection (at age 5 *n* = 556; at age 11 *n* = 121). Lastly, we also dropped twins and triplets (age 5 *n* = 162; age 11 *n* = 120) and cohort members for whom the mother was not the main interviewee at Sweep 1 (age 5 *n* = 1; age 11 *n* = 3). These exclusions reduced the analytical sample to 11 965 (78%) at 5 years, and 9384 (71%) at 11 years.

### Statistical analyses

We used multinomial logistic regression to estimate income gradients in child overweight and obesity whereby non-overweight children were the reference group. Models were run jointly for boys and girls and adjusted for gender of the child. The analyses were not stratified by gender since exploratory analyses (not shown) did not reveal differences for boys and girls in the role that risk factors had in attenuating income inequalities in children’s adiposity. In models estimated at age 11, we included a control variable for whether girls and boys exhibited any sign of pubertal changes (menarche, hair on body or breast growth for girls; voice change, hair on body or facial hair for boys).^34^^,^^35^ The baseline model shows the unadjusted association between income and overweight/obesity. Then we revealed the role that each category of risk factors had in attenuating income gradients in child overweight and obesity by adjusting for each category of risk factors separately: model 1 adjusts for maternal health behaviours; model 2 for markers of physical activity and sedentary behaviours and model 3 for dietary markers. In order to assess to what extent income inequalities were attenuated when all the risk factors were considered, Model 4 adjusts for all categories of risk factors simultaneously.

We assessed whether income inequalities widened significantly across ages by investigating whether poorer children were more likely to experience upward movements and less likely to experience downward movements across weight categories between age 5 and 11. To do so, we created two binary variables. One variable indicating whether the child transitioned from being normal weight at age 5 to being overweight/obese at age 11 or from being overweight to obese; the other binary variable indicating whether a child transitioned from being obese at age 5 to being overweight/normal weight at age 11 or transitioned from being overweight to normal weight. Using these binary indicators as dependent variables in a logistic regression model, we estimated income gradients in/upwards/downward movements with adjustment for the gender of the child and the child’s weight category at age 5 (normal weight, overweight, obese). Then, to investigate which category of risk factors measured at age 5 promoted or protected against these changes across income groups, we adjusted for each set of risk factors separately and, lastly, we estimated a model adjusting for all risk factors simultaneously. We focused on risk factors measured at age 5 in order to establish a temporal order between exposure to family health behaviours, routines and environmental risk factors, and children’s experiences of (upward or downward) weight changes. Adding to the models age 11 risk factors did not change our findings (results not reported). The logistic regression models were estimated on a sub-sample given that only children who were at risk of experiencing upward (normal weight or overweight children at age 5) or downward (obese or overweight children at age 5) movements were included in these analyses.

All analyses were conducted in Stata version 13. We used weights to account for non-response and overrepresentation of disadvantaged and ethnically diverse areas and the survey command to account for the clustering of samples within strata in the MCS. The model results are reported as relative risk ratios or odds ratios with 95% confidence intervals.

## Results

Table 1 shows that the prevalence of obesity was considerably higher among children in the poorest quintile compared with their peers in the top income quintile (6.6 vs. 3.5% at age 5; 7.9 vs. 2.9% at age 11). At age 5, there was no evidence of an income gradient in child overweight, but by age 11 differences between children from poorer compared with richer families had emerged (20.2 vs. 16.5%). Testing for linear trend shows that differences by income quintiles were statistically significant for obesity at age 5, and both overweight and obesity at age 11.

**Table 1:** Average % of overweight/obesity and description of risk factors by income quintiles at age 5 (*n* = 11965) and 11 (*n* = 9384)

	Age 5							Age 11						
	Income quintiles							Income quintiles						
	Bottom	Second	Third	Forth	Top	*P* values test for linear trend	Average	Bottom	Second	Third	Forth	Top	*P* values test for linear trend	Average
Child overweight	14.9	14.1	14.5	17.0	14.3	0.50	15.0	20.2	21.8	23.0	19.5	16.5	0.00	20.1
Child obesity	6.6	5.7	5.5	4.7	3.5	0.00	5.1	7.9	8.8	6.2	3.8	2.9	0.00	5.8
Mother's health behaviours														
Smoked during pregnancy	44.2	31.9	23.5	13.6	7.3		23.1	49.3	38.2	24.2	12.3	5.9		24.8
Introduction to solid foods before 4 months	34.6	36.2	33.9	29.4	23.3		31.2	35.3	35.6	33.6	29.6	23.6		31.4
No breastfeeding	45.9	37.7	28.4	23.6	13.6		29.0	57.8	43.3	34.6	19.8	12.2		32.3
Breastfeeding up to 2 months	27.8	30.3	31.3	29.4	26.5		29.0	25.3	31.5	29.0	29.1	25.7		28.2
Breastfeeding up to 4 months	9.4	11.0	13.0	14.3	18.3		13.4	6.4	9.2	11.0	15.0	18.6		12.4
Breastfeeding over 4 months	17.0	21.0	27.4	32.7	41.5		28.5	10.5	16.0	25.4	36.1	43.4		27.2
Child physical activity and sedentary behaviours														
Sport/exercise < 1 a week	69.3	59.1	43.9	32.6	21.8		44.1	39.5	34.0	23.5	17.0	13.5		24.8
Sport/exercise 1/2 a week	27.1	35.0	47.3	54.6	59.8		45.7	39.9	43.3	44.3	42.5	39.9		42.0
Sport/exercise 3+ a week	3.7	5.8	8.7	12.8	18.4		10.2	20.7	22.7	32.2	40.6	46.6		33.2
Active playing with a parent <1 a week	32.5	25.9	17.2	14.7	10.6		19.6	56.6	52.9	47.4	40.7	36.5		46.3
Active playing with a parent 1/2 a week	33.7	34.5	40.3	38.3	40.6		37.7	27.2	30.1	33.2	39.1	40.7		34.4
Active playing with a parent 3+ a week	33.8	39.5	42.5	47.0	48.8		42.7	16.2	17.0	19.3	20.2	22.8		19.3
TV less than 1 h on weekday	16.6	18.3	18.7	21.2	31.3		21.5	13.9	12.5	15.0	17.3	21.5		16.2
TV 1 h to under 3 h on weekday	61.4	64.2	67.8	66.7	60.6		64.2	65.4	69.9	70.0	69.2	66.6		68.3
TV 3 h or more on weekday	22.1	17.6	13.5	12.1	8.1		14.3	20.7	17.6	15.1	13.5	11.9		15.5
PC less than 1 h on weekday	69.0	73.7	78.0	80.4	86.5		77.9	47.7	49.3	53.9	55.2	60.3		53.6
PC 1 h to under 3 h on weekday	25.8	22.7	19.6	17.6	12.3		19.3	43.4	42.1	40.4	39.7	34.7		39.9
PC 3 h or more on weekday	5.2	3.6	2.4	2.0	1.2		2.8	8.9	8.6	5.6	5.1	5.1		6.5
Bedtime Age 5: before 7/Age 11: before 8	22.2	21.4	21.3	21.6	20.1		21.3	4.0	2.6	2.2	2.2	1.3		2.4
Bedtime Age 5: 7–7:59/Age 11: 8–8:59	23.5	29.6	35.4	38.6	42.5		34.4	28.3	27.6	33.2	36.8	40.5		33.6
Bedtime Age 5: 8–8:59/Age 11: 9–9:59	28.7	32.1	30.2	29.5	30.8		30.3	45.4	50.4	50.1	49.6	47.5		48.7
Bedtime 9 or later/Age 11: 10 or later	9.6	5.7	3.7	3.5	2.3		4.8	7.7	6.8	6.2	4.1	3.5		5.6
Bedtime not regular	16.0	11.2	9.4	6.7	4.4		9.3	14.7	12.7	8.2	7.3	7.1		9.8
Playground Age 5: child taken weekly/Age 11: availability in the area	66.3	60.5	61.1	58.5	60.1		61.2	90.7	91.7	93.2	94.8	96.0		93.4
Use of bike in the area (Age 11 only)	N/A							63.5	65.0	68.7	68.9	70.6		67.5
Child's diet														
Portions of fruit per day: 1 or none	29.0	23.5	19.0	14.9	10.7		19.0	41.4	36.4	32.1	26.1	18.9		30.4
Portions of fruit per day: 2	31.1	29.2	27.5	26.6	21.4		26.9	24.9	26.9	26.1	25.9	23.5		25.5
Portions of fruit per day: 3+	39.9	47.3	53.5	58.5	67.9		54.1	33.8	36.6	41.8	47.9	57.6		44.1
Skipping breakfast	11.4	8.4	5.9	4.5	3.5		6.5	22.4	18.8	11.9	9.8	6.4		13.4
Sweet drinks consumption	24.0	22.3	21.4	17.5	13.0		19.4	42.0	34.3	30.8	26.2	22.9		30.8
Mother BMI (mean)	25.3	25.8	25.3	24.9	24.3		25.1	26.8	26.2	26.2	25.3	24.8		25.8
Female child	50.1	49.0	49.3	47.8	50.0		49.2	48.4	47.6	48.3	47.9	49.6		48.4
Family income quintiles	17.7	18.9	20.5	21.1	21.8		20.0	17.1	19.9	20.2	20.9	21.8		20.0
Puberty	N/A							34.2	38.4	37.2	31.5	31.8		34.5

Figures are percentages unless otherwise specified

Note: all estimates are weighted and adjusted for design effects.

Table 1 also shows that there were striking income gradients in the distribution of risk factors. Children in the lower income quintiles were more likely to have mothers who smoked during pregnancy, were not breastfed or breastfed for a shorter duration, were introduced to solid food earlier; they were less likely to do sports, to engage in active playing with a parent, to be living in an area with a playground and to use a bike in the area, spent more time watching TV and using a PC, and they tended to have later or irregular bedtimes; they were also less likely to be eating fruit, to have breakfast every day and their mothers had higher average BMIs. In contrast, at age 5 children in the lower income quintiles were more likely to be taken to the playground at least weekly.

### Which risk factors might explain income inequalities?

Table 2 shows selected results from the multinomial logistic regression models at age 5 (*n* = 11 965): the reported risk of being overweight and obese relative to the risk of being non-overweight (results for the risk factors are available upon request). In the baseline model, poorer children experienced significantly higher relative risk of being obese than children in the top income quintile (2.0 95% CI: 1.4–2.8). Physical activity and dietary risk factors appeared most important in attenuating inequalities. After adjustment for all risk factors in the final model, income inequalities were substantially reduced. On adjustment for all risk factors in Model 4, children in the bottom income quintile had a reduction in the relative risk of obesity of around 60%. There was no evidence of an income gradient in the relative risk of being overweight at age 5.

**Table 2:** Relative risk ratios (95% confidence interval) of overweight and obesity at age 5 by income quintiles with additional adjustments for risk factors at age 5 (*n* = 11 965)

	Multinomial logistic model results				
	Baseline model^a^	Model 1: Baseline model + mother's health behaviours^b^	Model 2: baseline model + physical activity markers^c^	Model 3: baseline model + dietary markers^d^	Model 4: baseline model + all risk factors^e^
Overweight age 5					
Top	Reference	Reference	Reference	Reference	Reference
Fourth	1.3(1.0–1.5)	1.2(1.0–1.4)	1.2(1.0–1.5)	1.2(1.0–1.5)	1.2(1.0–1.4)
Third	1.0(0.9–1.3)	0.9(0.8–1.1)	1.0(0.8–1.2)	1.0(0.8–1.2)	0.9(0.7–1.1)
Second	1.0(0.8–1.2)	0.9(0.7–1.1)	1.0(0.8–1.2)	0.9(0.8–1.1)	0.8(0.7–1.0)
Bottom	1.1(0.9–1.3)	0.9(0.8–1.1)	1.0(0.9–1.3)	1.0(0.9–1.2)	0.9(0.7–1.1)
Obesity age 5					
Top	Reference	Reference	Reference	Reference	Reference
Fourth	1.4(1.0–2.1)	1.4(0.9–2.0)	1.4(0.9–2.0)	1.3(0.9–1.9)	1.3(0.9–1.9)
Third	1.6(1.1–2.3)	1.5(1.0–2.2)	1.5(1.0–2.1)	1.4(1.0–2.0)	1.3(0.8–1.9)
Second	1.7(1.2–2.4)	1.5(1.0–2.2)	1.4(1.0–2.1)	1.3(0.9–1.9)	1.1(0.8–1.7)
Bottom	2.0(1.4–2.8)	1.7(1.2–2.5)	1.6(1.1–2.3)	1.6(1.1–2.3)	1.3(0.9–2.0)

All estimates are weighted and adjusted for design effects.

a Adjusted for child's gender.

b Adjusted for child's gender, mother smoking during pregnancy, length of breastfeeding, introduction to solid foods before 4 months.

c Adjusted for child's gender, frequency of sport per week, frequency of active playing with a parent per week, frequency of TV watching, frequency of PC use, bedtime, frequency child is taken to the playground.

d Adjusted for child's gender, fruit portion per day, eating breakfast every day, maternal BMI and sweet drinks consumption.

e Adjusted for child's gender and all risk factors.

Table 3 shows selected results of the multinomial regression models at age 11 (*n* = 9384). In the unadjusted models, inequalities appeared more pronounced compared to age 5. Children in the bottom income quintile had 3.0 (95% CI: 2.0–4.5) increased relative risk of being obese at age 11. At age 11, children in the second income quintile had the highest relative risk of being obese (3.4 95% CI: 2.2–5.1), but, as revealed by the overlap in the confidence intervals, differences between the bottom and second income quintiles were not statistically significant. For children in the bottom quintile, adjusting for dietary markers did most to attenuate inequalities.

**Table 3:** Relative risk ratios (95% confidence interval) of overweight and obesity at age 11 by income quintiles with additional adjustments for risk factors at age 11 (*n* = 9384)

	Multinomial logistic model results				
	Baseline model^a^	Model 1: Baseline model + mother's health behaviours^b^	Model 2: baseline model + physical activity markers^c^	Model 3: baseline model + dietary markers^d^	Model 4: baseline model + all risk factors^e^
Overweight age 11					
Top	Reference	Reference	Reference	Reference	Reference
Fourth	1.3(1.1–1.5)	1.2(1.0–1.5)	1.2(1.0–1.5)	1.2(1.0–1.4)	1.2(1.0–1.4)
Third	1.5(1.3–1.8)	1.5(1.2–1.8)	1.4(1.2–1.7)	1.4(1.1–1.6)	1.3(1.1–1.6)
Second	1.5(1.2–1.8)	1.4(1.2–1.7)	1.3(1.1–1.6)	1.3(1.1–1.6)	1.2(1.0–1.4)
Bottom	1.4(1.1–1.6)	1.3(1.1–1.6)	1.2(1.0–1.5)	1.1(0.9–1.3)	1.0(0.8–1.2)
Obese age 11					
Top	Reference	Reference	Reference	Reference	Reference
Fourth	1.4(0.9–2.1)	1.3(0.9–1.9)	1.3(0.9–1.9)	1.3(0.8–1.9)	1.2(0.8–1.7)
Third	2.4(1.6–3.6)	2.0(1.3–3.1)	2.1(1.4–3.1)	1.8(1.2–2.8)	1.5(1.0–2.3)
Second	3.4(2.2–5.1)	2.7(1.8–4.2)	2.6(1.7–4.0)	2.6(1.7–3.9)	1.9(1.2–2.9)
Bottom	3.0(2.0–4.5)	2.3(1.5–3.6)	2.3(1.5–3.5)	2.0(1.3–3.1)	1.4(0.9–2.2)

All estimates are weighted and adjusted for design effects.

a Adjusted for child's gender.

b Adjusted for child's gender, mother smoking during pregnancy, length of breastfeeding, introduction to solid foods before 4 months.

c Adjusted for child's gender, frequency of sport per week, frequency of active playing with a parent per week, frequency of TV watching, frequency of PC use, bedtime, frequency child is taken to the playground.

d Adjusted for child's gender, fruit portion per day, eating breakfast every day, maternal BMI and sweet drinks consumption.

e Adjusted for child's gender and all risk factors.

At age 11, we also observed an income gradient in the relative risk of being overweight. Children in the bottom income quintile had 1.4 (95% CI: 1.1–1.6) increased relative risk of being overweight compared to their peers in the highest income quintile. On adjustment for all risk factors in Model 4, the relative risk of overweight for children in the bottom income quintile was fully attenuated.

### Upward and downward movements across weight categories and which risk factors might explain them

Table 4 shows that children, who at age 5 were either normal weight or overweight, in the bottom, second and third income quintiles were significantly more likely to experience upward movement across weight categories than children in the top quintile (results for the risk factors are available upon request). Adjustment for physical activity and dietary markers did most to attenuate inequalities. On adjustment for all risk factors, children in the lowest income quintile compared to children in the highest income quintile had a reduction in the odds of moving up a weight category of around 55%. Children in the second income quintile were less likely to experience downward movements across weight categories, but the results fail to show a clear income gradient in downward movements across weight categories for children who were either obese or overweight at age 5. Nonetheless, these results are informative because they suggest that similar risk factors were protective against upward movements and promoted downward movements (results available upon request). An earlier bedtime and fruit consumption more than 3 times a day were negatively associated with upward movements and positively associated with downward movements across weight categories. Mother’s smoking during pregnancy, introduction to solid foods before 4 months and mother’s BMI were positively associated with upward movements and negatively associated with downward movements across weight categories. Adjusting for risk factors measured at age 11 resulted in smoking no longer being statistically significant but the rest of the coefficients remained largely unchanged (results available upon request).

**Table 4:** Average prevalence and odds ratios (95% confidence interval) of upward and downward movements between age 5 and 11 by income quintiles

		Multinomial logistic model results				
	Average %	Baseline model^a^	Model 1: Baseline model + mother's health behaviours^b^	Model 2: baseline model + physical activity markers^c^	Model 3: baseline model + dietary markers^d^	Model 4: baseline model + all risk factors^e^
Moving up a category between age 5 and 11 (*n* = 8852)*						
Top	10.5 (*n* = 200)	Reference	Reference	Reference	Reference	Reference
Fourth	12.1 (*n* = 242)	1.1(0.9–1.4)	1.1(0.8–1.4)	1.1(0.8–1.4)	1.0(0.8–1.3)	1.0(0.8–1.3)
Third	16.3 (*n* = 313)	1.6(1.3–2.0)	1.5(1.2–1.9)	1.5(1.2–1.8)	1.4(1.2–1.8)	1.3(1.1–1.6)
Second	19.6 (*n* = 336)	2.0(1.6–2.5)	1.8(1.4–2.3)	1.8(1.4–2.2)	1.7(1.3–2.1)	1.5(1.1–1.9)
Bottom	18.5 (*n* = 295)	1.9(1.5–2.3)	1.6(1.3–2.1)	1.5(1.2–2.0)	1.5(1.2–1.9)	1.3(1.0–1.7)
Moving down a category between age 5 and 11 (*n* = 1852)^**^						
Top	43.7 (*n* = 155)	Reference	Reference	Reference	Reference	Reference
Fourth	43.4 (*n* = 181)	1.0(0.7–1.4)	1.0(0.8–1.4)	1.0(0.8–1.4)	1.1(0.8–1.5)	1.1(0.8–1.5)
Third	42.8 (*n* = 157)	1.0(0.7–1.4)	1.0(0.7–1.5)	1.0(0.7–1.4)	1.2(0.8–1.7)	1.2(0.8–1.7)
Second	32.0 (*n* = 115)	0.6(0.4–0.9)	0.7(0.5–1.0)	0.7(0.5–1.0)	0.8(0.6–1.2)	0.9(0.6–1.3)
Bottom	43.5 (*n* = 134)	1.0(0.7–1.5)	1.1(0.7–1.7)	1.2(0.8–1.7)	1.4(0.9–2.0)	1.5(1.0–2.3)

These models were run a sub-sample of children who were at risk of experiencing upward to downward movements across weight categories between age 5 and 11:.

* Children who at age 5 where either normal weight or overweight.

** Children who at age 5 where either obese or overweight. All estimates are weighted and adjusted for design effects.

a Adjusted for child's gender and puberty.

b Adjusted for child's gender, puberty, mother smoking during pregnancy, length of breastfeeding, introduction to solid foods before 4 months.

c Adjusted for child's gender, puberty, frequency of sport per week, frequency of active playing with a parent per week, frequency of TV watching, frequency of PC use, bedtime, frequency child is taken to the playground.

d Adjusted for child's gender, puberty, fruit portion per day, eating breakfast everyday, maternal BMI and sweet drinks consumption.

e Adjusted for child's gender, puberty and all risk factors.

## Discussion

We show, consistent with prior studies,^10^ stark income inequalities in the risk of obesity throughout childhood, the emergence of inequalities in the risk of overweight by age 11 and that inequalities became more pronounced between age 5 and 11. After adjustment for risk factors, inequalities between the bottom and top income quintiles were attenuated by at least 50% and were largely no longer statistically significant. Multiple family health behaviours and environmental risk factors were relevant when attempting to explain income inequalities in overweight and obesity amongst children. The results suggest that both markers of physical activity and diet at ages 5 and 11 were particularly relevant in attenuating inequalities throughout childhood. However, maternal health behaviours in early childhood were relevant too as on their own in the model, they attenuated inequalities in child obesity between the bottom and top income quintiles by around 20%. This perhaps indicates that pathways underpinning inequalities in child adiposity begin early in life and are cumulative over the life course. The influence of each set of risk factors was relatively similar across childhood and some markers were relevant at both ages, such as the mother’s BMI, early bedtimes and watching TV less than 1 h per weekday. However, other markers were more relevant at age 5 or 11. For example, skipping breakfast was related to the risk of overweight and obesity, and fruit consumption was related to the risk of overweight at age 5, but these factors played a rather minor role at age 11. In contrast, doing sport more than three times a week played a more important and protective role at age 11 than age 5.

The magnitude of income inequalities in child obesity and overweight grew between ages 5 and 11. Poorer children were more likely to experience upward movements across weight categories than richer ones. These findings suggest that efforts to curb the increasing prevalence of obesity, particularly amongst disadvantaged children, should start early in life. Intervening in the early years when the family environment has more profound influences on children’s healthy development has the potential to be particularly effective—setting children onto ‘healthy’, or at least healthier, adiposity trajectories. Importantly, the results reveal that similar risk factors protected against and promoted, respectively, upward and downward movements across weight categories. This might point to the importance of promoting (e.g. an earlier bedtime, fruit consumption more than 3 times a day) or discouraging (e.g. smoking during pregnancy, early introduction to solid foods and high maternal BMI) these behaviours in an effort to curb further increases in the prevalence of overweight and obesity amongst poorer children.

There are several government initiatives e.g. the Change4life programme,^1^ which aim to prevent and address body fat gain amongst children in the UK. Generally, existing policies support healthy living in the family environment by promoting—amongst adults and children—healthy eating (e.g. ‘5-a-day’) and exercise (e.g. ‘10 000 steps a day’). Our findings support the development of horizontal prevention strategies aimed at tackling a wider set of risk factors from multiple domains including physical, sedentary and dietary behaviours. However, the evidence on effective policy strategies that reduce or eliminate disparities in children’s adiposity is not yet conclusive.^36^ Greater efforts are needed to investigate which interventions might contribute to reduce the prevalence of child overweight and obesity. In particular, more research and evaluations are needed to understand which changes in family and children’s routines might contribute to a reduction in socioeconomic inequalities in children’s adiposity at different ages and their widening across childhood. Based on the findings of this study, interventions focussing on family physical activity and ‘healthier diets’ could be of benefit, for instance these could include family sport days, distribution of gym passes and cookery classes.

### Strengths and limitations

We used data from the Millennium Cohort Study which provides a large nationally representative sample to investigate inequalities in child obesity and overweight across income quintiles. We were also able to begin exploring the mechanisms behind income disparities in child obesity and overweight but with some limitations. First, our models were unable to fully explain income inequalities in children’s adiposity suggesting that other risk factors should be considered in future work. Second, the results did not allow for a conclusion regarding causality between risk factors and income inequalities in overweight and obesity. Third, maternal BMI was included in the analyses in the category ‘dietary environment’, but it may also reflect genetic as well as shared environment. Given that maternal BMI was the only factor consistently and significantly associated with child overweight and obesity, we ran some sensitivity tests by including the rest of the risk factors related to diet on their own (i.e. without mother’s BMI). The results (not shown) reveal that maternal BMI plays a substantial role, yet modelling other dietary factors on their own partially attenuates the unadjusted differences. Fourth, BMI is an imperfect measure of adiposity as it does not distinguish between fat and lean mass.^37^ We conducted sensitivity analyses by categorizing children as overweight and obese using body fat at age 11 (not measured at age 5). The results (available on request) were largely consistent with those obtained using BMI. Fifth, health behaviours were reported by the mother. Parental reports might be more suitable for younger children, but parents are frequently unaware of the behaviours of 11-year-olds who spend considerable time beyond purview. Sixth, it could be that children who experienced upward movements across weight categories between age 5 and 11 were already on the cusp of their weight categories in the previous period and differed from children who were on the lower end of healthy weight/overweight at age 5. To assess whether this could be the case, in additional analyses (not shown), we compare average BMI at age 5 of children who experienced/did not experience upward movements across weight categories. Results reveal that although children who experienced upward movements had higher BMI levels at age 5, differences between the two groups were not large (approximately 0.2 SD of BMI) suggesting that those who moved up a weight category were not necessarily on the cusp of their weight category at age 5. Finally, as in any longitudinal study, missing data because of loss to follow-up could bias the results. In order to account for this, we used non-response weights but we may not have been able to fully adjust for sample attrition.

### Future work

As the processes involved in the development of fat gain in children involve social, environmental and biological factors, future work should be directed at more closely examining different typologies of risk factors and their interaction. More work is needed to establish whether there would be a reduction in inequalities and in the widening of the inequality gap across childhood if poorer families and their children adopted healthier behaviours.
